# Transfer of *bla*TEM gene between *Salmonella* and *Escherichia coli* under processing conditions of animal products: influence of a copper(II) complex

**DOI:** 10.3389/fmicb.2025.1676649

**Published:** 2025-11-07

**Authors:** Rosanne Aparecida Capanema Ribeiro, Micaela Guidotti-Takeuchi, Carolyne Ferreira Dumont, Ana Beatriz Garcez Buiatte, Bárbara de Araújo Brum, Thais Jansen Martins, Luana Munique Sousa Ramos, Wendell Guerra, Richard Costa Polveiro, Roberta Torres de Melo, Daise Aparecida Rossi

**Affiliations:** 1 Laboratório de Biotecnologia Animal Aplicada, Faculdade de Medicina Veterinária, Universidade Federal de Uberlândia, Uberlândia, Brazil; 2 Instituto Federal de Educação, Ciência e Tecnologia Farroupilha, Santa Maria, Brazil; 3 Instituto de Química, Universidade Federal de Uberlândia, Uberlândia, Brazil

**Keywords:** bacterial conjugation, whey, chicken juice, copper(II) complex, *bla*TEM

## Abstract

The high prevalence of infections caused by contaminated food, coupled with growing antimicrobial resistance, especially through horizontal gene transfer, is a challenge for public health worldwide. It is possible that this situation is intensified in the presence of by-products from animal product processing industries. In view of this, we investigated the horizontal transfer of the *bla*TEM gene from *S.* Heidelberg to *E. coli* J53 AzR, in the absence and presence of whey (WH) and chicken juice (CJ) in: (i) liquid medium for 3 h under agitation; (ii) solid medium overnight; (iii) liquid medium *overnight* and the influence of the copper(II) complex Lu54 in mitigating this transfer. The first protocol showed the highest relative conjugation frequency (RCF) of 2.23% in the absence of supplements and increased by three and four orders of magnitude in the presence of CJ and WH and was selected for treatment with Lu54. In *solid/overnight*, there were RCFs of less than 1%, while the *liquid/overnight* medium showed RCFs higher than the first protocol only in WH. The presence of WH acidified the medium, which resulted in higher RCF. Lu 54 reduced RCF from 2.2 to 0.3%, 8.2 to 1.7% and 6.2 to 0.9%, respectively, for the tests without by-products and with WH and CJ. In addition, the genomes were sequenced to map the *bla*TEM gene and *β*-lactamase families in transconjugants. The results showed that three plasmids containing *bla*TEM were detected in the controls and the same gene was not identified in the treatments, suggesting plasmid loss induced by the copper(II) complex (Lu54). The results prove that WH and CJ increase the frequency of conjugation in liquid media, and the Lu54 complex is a promising alternative to mitigate conjugation and, consequently, the spread of antimicrobial resistance, especially in milk and meat processing industries.

## 1 Introduction

*Salmonella* Heidelberg and *Escherichia coli* are pathogens of worldwide importance, frequently related to foodborne outbreaks (Elhariri et al., 2020; CDC, 2022). The clinical manifestations associated with the consumption of food contaminated by these bacteria are especially characterized by enteric symptoms, but depending on the host–parasite relationship, complications can be observed, even fatal ones (EFSA, 2020; Abu Daher et al., 2019).

In fact, epidemiological data from infants in the United States between 1968 and 2015 revealed that *S*. Heidelberg accounted for 29% (1954/6767) of bacteremia and 18% (65/371) of meningitis cases in this age group, with hospitalization rates exceeding 70% and case fatality rates reaching 4% in meningitis (Schmidt et al., 2023). In Australia, a multistate outbreak in 2018–2019 across five jurisdictions resulted in a hospitalization rate of 36% (22/59), highlighting the moderate severity and invasiveness of the strain involved (Kerr et al., 2022), and a prolonged outbreaks linked to backyard poultry, approximately 33% of patients with available clinical data required hospitalization, highlighting the severity of these infections (CDC, 2024). More recent evidence from a prolonged outbreak associated with poultry in the United States (2013–2014) indicated that *S.* Heidelberg was responsible for 634 infections, with hospitalization and invasive disease rates of 38 and 15%, respectively. RNA sequencing of nine isolates from the outbreak revealed increased heat tolerance, enhanced biofilm formation, and transcriptional priming of multidrug efflux and virulence genes, indicating an enhanced ability to survive processing stress and cause disease (Etter et al., 2019). Together, these findings highlight the significant health impact caused by S. Heidelberg in vulnerable groups.

In addition to the health hazards and economic losses associated with infectious diseases due to the considerable morbidity and mortality rates, there is growing concern about an additional problem, the increase in antimicrobial resistance (AMR), which makes treatment and control more difficult (Lamichhane et al., 2024). Although AMR occurs naturally in bacteria, some factors can aggravate the problem and contribute to its spread. These include the inappropriate use of antimicrobials, poor regulation and oversight of the use of veterinary and human antibiotics, which exert selection pressure on resistant strains, extending even to other antimicrobials not involved in the initial challenge (Reygaert, 2018; Murray et al., 2022; MAPA, 2022). As a consequence, data presented by the US Centers for Disease Control and Prevention (2019) show that more than 2.8 million antibiotic-resistant infections occur in the United States each year, resulting in more than 35,000 deaths (CDC, 2019).

One of the antibiotics widely used to treat various infections by Gram-positive and Gram-negative bacteria is the beta-lactam ampicillin, which acts by inhibiting the synthesis of the bacterial cell wall, causing it to weaken and cell lysis (NCBI, 2022). Due to indiscriminate use, AMR to this drug is growing, especially due to the presence of *β*-lactam resistance genes, such as *bla*(TEM; Elhariri et al., 2020; Srichumporn et al., 2022; Delmani et al., 2021).

Among the factors that contribute to the spread of AMR is horizontal gene transfer, in which there is temporary or permanent acquisition of genetic material from another microorganism. Transfer can occur through transformation, transduction and conjugation (Reygaert, 2018). Conjugation involves the transfer of plasmids through direct contact between a donor and recipient bacterium, and is considered the major mechanism of horizontal gene transfer (HGT; Neil et al., 2021). This transfer begins in the cytoplasm, where the relaxosome binds to the transfer origin (*oriT*) present in the DNA and then the coupling protein (T4CP) guides the *relaxosome-oriT* complex to the type IV T4SS secretion system present in the membrane, through which the DNA strand leaves the donor bacterium and passes to the recipient via the *pili* (Ilangovan et al., 2015; Lee and Grossman, 2007).

Plasmids containing AMR genes can spread rapidly between different bacterial strains, species or genera, which leads to the global dissemination of various genes responsible for resistance to different classes of drugs (Von Wintersdorff et al., 2016; Huddleston, 2014). Gene transfer can also endow the recipient bacterium with new metabolic capabilities, facilitating its adaptation to diverse ecological niches (Sultan et al., 2018).

Different situations can influence the frequency of conjugation, such as cell density, solid or liquid substrate, temperature, presence of nutrients, among others (Alderliesten et al., 2020; Shintani et al., 2019), but the strategies for sharing genes between bacteria are still little known in the food production chain. This prompts questions about the influence of by-products present in animal product processors, such as chicken juice and whey, on bacterial conjugation rates. It is known that chicken juice stimulates multiplication and is capable of positively interfering with bacterial virulence mechanisms, such as biofilm formation (Melo et al., 2017), but its action is not known when it comes to gene transfer. There are also no studies in the literature investigating whether whey can exert any influence on this phenomenon.

As a way of mitigating the dangers of resistance to available antimicrobials, it is important to look for new alternatives, and among the options are copper complexes, which have demonstrated antimicrobial properties (Krasnovskaya et al., 2020; Zalevskaya and Gur’eva, 2021). Ott and Mellata (2024) demonstrated that short-chain fatty acids inhibit the conjugation of resistant plasmids between *Escherichia coli in vitro* and in chicken tissue explants, suggesting applicability in animal processing environments. Another study showed that extracts of natural products and pure compounds reduce the conjugation of *Enterobacterales* plasmids, including plasmids encoding broad-spectrum *β*-lactamases or carbapenemases (“IncK,” “IncFII,” etc.), although the magnitude of the reduction is still modest (Alav et al., 2024). In addition, Jia et al. (2024) identified that azidothymidine (AZT), a drug used as a reverse transcriptase inhibitor for HIV, has a broad inhibitory effect on the transfer of plasmids with antibiotic resistance genes (ARG) between clinically relevant species, acting on energy metabolism, bacterial secretion system, and membrane potential; this finding points to synthetic alternatives with potential for practical use.

In this context of searching for innovative compounds to mitigate the spread of resistance, copper stands out. Copper is an essential element, it acting as a cofactor in different enzymatic reactions, energy metabolism, respiration, cellular DNA synthesis, it as a powerful antioxidant that has demonstrated, for example, efficacy in inhibiting *S. typhimurium* when in synergism with other antibiotics, which makes its use in the development of antimicrobial drugs interesting (Stern, 2010; Monteiro et al., 2022). Regardin the mechanism of action, copper complexes promote the generation of reactive oxygen species (ROS), disrupt enzyme activity, induce DNA damage, and compromise cell membrane integrity (Ngece et al., 2025). These effects explain why copper have emerged as promising candidates, since factors such as the nature of the ligands, coordination geometry, lipophilicity, and electron donor systems significantly modulate their antimicrobial potency and selectivity (Salah et al., 2021; Ngece et al., 2025). In this scenario, the copper(II) complex Lu54 (Takeuchi et al., in press) emerges as a relevant proposal, as it can promote greater selectivity, act as a conjugation inhibitor, and maintain activity against resistant strains.

We aimed to evaluate the influence of chicken juice and whey on the transfer of the *bla*TEM gene between *Salmonella* Heidelberg and *E. coli* J53 AzR under different conditions, such as solid and liquid media at different incubation times, and to determine the effectiveness of the copper(II) complex Lu54 [Cu(Clmp)(mTFpy)](PF_6_)_2_ in mitigating recombination rates by conjugation.

## 2 Materials and methods

chicken juice (CJ) and whey (WH). chicken juice was obtained as described by Brown et al. (2014), with modifications. Chicken exudate was collected from frozen commercial chicken thawed at room temperature for approximately 24 h. Subsequently, the chicken juice was centrifuged at least three times at 14,000 rpm for 10 min at 4 °C to eliminate large particles, and the supernatant was sterilized by filtration through a membrane with a pore size of 0.22 μm (Kasvi®, Paraná, Brazil). Similarly, whey collected from Minas frescal cheese was prepared and sterilized. Both supplements were stored at −20 °C.

Copper(II) complex Lu54. The copper(II) complex of general formula [Cu(clmp)(mtfpy)](PF_6_)_2_ (clmp = 4-chloro-N-(pyridine-2-methylene)aniline and mtfpy = 4′-(4-methylphenyl)-2,2′:6′,2″-terpyridine), herein designated Lu54, was produced and fully characterized at the Institute of Chemistry of the Federal University of Uberlândia. In this complex, the Cu(II) ions adopt a square pyramidal geometry, involving the three nitrogen atoms of the mTFpy ligand and the two nitrogen atoms of Clmp. Furthermore, in the solid state, two PF_6_^−^ anions act as counter ions (Takeuchi et al., n.d.).

Determination of the target concentration of Lu54. The minimal inhibitory concentration (MIC) of the copper(II) complex Lu54 was determined using the broth microdilution method (EUCAST, 2022). Briefly, concentrations of 500, 250, 125, 62.5 and 31.25 μg/mL were initially tested. The bacterial suspensions were prepared at a concentration corresponding to 0.5 on the MacFarland scale, inoculated into microplates containing Mueller-Hinton broth (Kasvi®, Paraná, Brazil) with the copper complex at different concentrations, and then incubated at 37 °C for 24 h. For the negative control, culture medium without added bacteria was used; and for the positive control, culture medium with added *S.* Heidelberg and *E. coli* J53 bacteria without added complex was used.

The wells containing concentrations lower than the MIC were quantified using the plate count method in order to verify the highest concentration of Lu54 that did not exert bactericidal activity against the *Salmonella* Heidelberg and *E. coli* strains. This was due to the need to select the highest concentration that would allow the cells to survive and thus assess its effect on conjugation.

All the tests were carried out in three replicates and three repetitions.

Bacterial conjugation. Three different conjugation protocols were tested and the one with the highest conjugation frequency was selected for the Lu54 copper complex tests.

In all protocols, the donor strain used was *Salmonella enterica* Heidelberg (SH), which has sexual *pili* and the plasmid *bla*TEM gene, which codes for resistance to the *β*-lactam class of antimicrobials, such as ampicillin. *S.* Heidelberg was previously isolated from broiler litter in 2018, characterized by MELO et al. (2021), and deposited in the strain bank of the Molecular Epidemiology Laboratory of the Faculty of Veterinary Medicine of the Federal University of Uberlândia. The J53AzR (J53) strain of *E. coli*, which is resistant to sodium azide and does not have the *bla*TEM gene, was used as a recipient and is sensitive to ampicillin.

The SH and J53 strains were reactivated in brain and heart infusion pre-enrichment broth (BHI, Sigma-Aldrich®, Missouri, United States) at 37 °C for a minimum period of 6 h, until visual growth is observed due to turbidity and then individually replated on Tripitic Soy Agar (TSA - Biokar®, Allone, France), incubated at 37 °C for 18 h (Mo et al., 2017).

The conjugation protocols tested followed the methodologies proposed by Rodriguez & Fernandez (Rodriguez-Grande and Fernandez-Lopez, 2020), with adaptations. Briefly, approximately five colonies from each strain (donor and recipient) were selected and inoculated separately into sterile Falcon tubes containing 3 mL of Luria Bertani broth (LB-Neogen®, Michigan, USA) and incubated at 37 °C on an orbital shaker rotating at 60 rpm for approximately 3 h. After incubation, the absorbance (O. D.600 nm) was measured on a microplate reader (Perlong DNM-9602, Nanjing, China) to check whether the exponential phase had been reached (O. D.600 nm = 0.4 to 0.5).

The cultures of the donor and recipient strains were individually subjected to serial decimal dilution (10^−1^ to 10^−8^) on surface MacConkey agar (MC, Biokar®, Allone, France), enriched with 90 μg/mL ampicillin for SH growth and 160 μg/mL sodium azide for J53, in order to phenotypically express their resistance. The plates were incubated at 37 °C for 24 h, when the number of colonies was counted.

For conjugation protocol 1, SH and J53 were mixed (1:4 ratio) in a final volume of 1 mL. In parallel, the same process was carried out, but adding 5% of the WH or CJ (50 μL) to 190 μL of the donor strain and 760 μL of the recipient strain previously grown in LB. Finally, the tubes containing the cocultures, with and without the addition of WH and CJ, were incubated at 37 °C with slow agitation (20 rpm) for 3 h.

Conjugation protocol 2 used solid medium. Briefly, 0.22 micrometer cellulose ester membranes were placed on LB agar distributed in six-well cell culture plates. Next, 200 μL of the coculture previously obtained in conjugation 1, with and without the addition of 5% WH or CJ were added to the membrane. The plates were incubated at 37 °C *overnight.*

For conjugation protocol 3, the procedure was initially identical to conjugation protocol 1 and after 3 h of conjugation, a volume of 400 μL of cocultures, with and without the addition of WH or CJ, was transferred to 1,600 μL of LB broth in a sterile flask in order to renew the culture medium, followed by incubation at 37 °C *overnight* without stirring. The same procedure was carried out with LB medium supplemented with 5% WH or CJ, maintaining a 1:4 ratio.

All tests were carried out in triplicate and in three technical replicates.

Identification of transconjugants. The frequency of transfer of resistance to *β*-lactams was investigated by determining the acquisition of the *bla*TEM gene by phenotypic and molecular methods.

For phenotypic confirmation, after the conjugation assays, the cultures were subjected to serial decimal dilution (10^−1^to 10^−8^), sown on the surface of MacConkey agar (Kasvi®, Paraná, Brazil) supplemented with 90 μg/mL ampicillin and 160 μg/mL sodium azide (MCT), and incubated at 37 °C for 48 h.

In the case of conjugation 2, which used solid medium, phenotypic identification took place after submerging the membrane in 20 mL of saline solution to resuspend the cell mass, as well as serial dilution and seeding on MCT plates, followed by the procedures described above. The selection of the transconjugants was determined by the growth of colonies on the MCT plates, since only the strains of *E. coli* J53 AzR that have acquired the resistance gene *bla*TEM show resistance to both antimicrobials, in addition to the inhibition of growth of *S*. Heidelberg due to the presence of sodium azide.

Lu54 complex in conjugation frequency. After carrying out the three different conjugation protocols, the one with the highest conjugation frequencies was selected for treatment with 100 μL of the Lu54 complex. The concentration used was the pre-established one, lower than the MIC, in which we obtained no statistical difference with the controls, so as not to exert bactericidal activity.

Conjugation frequency. The efficiency of conjugation was measured by calculating the relative frequency of conjugation by the recipient (RCF), which is the ratio of the number of transconjugating colonies at the end of the conjugation process to the number of recipients at the start of the process, counted by the average number of repetitions of each treatment. Plates were selected for dilutions with growth between 25 and 250 colonies (Taniwaki et al., 2017). Calculations were carried out separately for conjugations with and without the addition of WH and CJ, and also for those treated with the Lu54 copper complex.


$$\mathrm{RCF}:\frac{\text{Mean number of transconjugating colonies}}{\text{Mean number of recipient colonies}}$$

Visualization in Scanning Electron Microscopy (SEM). The ultrastructure of conjugation was viewed by scanning microscopy, using a modified method (Brown et al., 2014) considering the growth conditions in Luria Bertani broth (LB-Neogen®) and conjugation at 37 °C for 3 h/20 rpm. After conjugation, samples were fixed on 1-cm2 (PU) slides (Habasit Cleandrive TM, Reinach, CH) and fixed with 2.5% glutaraldehyde and 2.5% paraformaldehyde in 0.1 M PBS buffer (pH 7.4) at 4 °C overnight. Subsequently, the fixative was removed, and the samples were washed with PBS buffer. The samples were post-fixed for 2 h with 1% osmium tetroxide and washed three times with PBS buffer. The dehydrated series was applied with ethanol solutions (30, 40, 50, 60, 70, 80, and 90% and then three times at 100%) for 20 min for each step. The samples were dried in a CPD (critical drying point) system (Leica EM CPD300; Leica, Wien, Austria) using liquid carbon dioxide as a transition fluid, coated with a 20-nm gold layer (SCD 050, Baltec), and exposed on a Zeiss Supra 55 FEG SEM operating at 5 kV. Images were captured with standard ZEISS SmartSEM.

pH evaluation. The pH assessment was carried out using a previously calibrated MS mPA210 pH meter (Tecnopon, São Paulo, Brazil) and took place in the liquid conjugation media (protocols 1 and 3), with the first measurement (0 h) carried out immediately after the donor and recipient were joined; the second (3 h), after conjugation 1 had finished; and the third the following day (*overnight*), after conjugation 3 had finished. All the procedures were carried out with and without the addition of WH and *CJ*.

The tests were carried out in triplicate and two technical replicates.

Whole genome sequencing (WGS) and pre-processing of the raw data. DNA extracted from *E. coli* using a commercial extraction kit (Wizard Genomic DNA Purification Kit—Promega) and the methodology described above was sent for whole-genome sequencing. Six samples were selected: three DNA samples from bacteria not treated with Lu54, one with added WH, one with CJ and one without WH and CJ; and three treated with Lu54, one with added WH, one with CJ and one without WH and CJ. The genomic DNA was sequenced using the MinION platform from Oxford Nanopore Technologies (ONT) with the Rapid Sequencing gDNA-Barcoding kit (SQK- RBK004) in a run time of 72 h. To prepare the libraries, 5–10 ng of DNA were used per sample (3 μL volume). The raw sequencing data was subjected to an initial quality assessment to check the size distribution and coverage of the *reads*, followed by the selection of *reads* with high integrity for the subsequent assembly stages. *Reads* were filtered by quality thresholds (Phred scores: Q > 10, Q > 15 and Q > 20) using Filtlong v0.2.1 (Wick, 2021a, b). The quality metrics and summaries were generated with NanoPlot v1.41.0 (Coster, 2018), and the removal of adapters was carried out with Porechop v0.2.4 (Wick et al., 2017).

Genome assembly, quality check and annotation of plasmids and *bla*TEM gene. The Raven 1.8.0 tool (Vaser and Šikić, 2021) was used to assemble the genomes using the *de novo* methodology. QUAST v.5.0.2 (Mikheenko et al., 2018) and CheckM v.1.1.3 (Parks et al., 2015) were used to assess the quality of the genomes. These tools were used to ensure that the genomes had >90% completeness, < 5% contamination,< 200 contigs and N50 > 40,000 bp. To confirm that the genomes belonged to the *Escherichia coli* species, we calculated the genome-wide *average nucleotide identity* (ANI) with the reference genome (ASM584v2—GenBank accession GCA_000005845.2) using FastANI v.1.32 (Jain et al., 2018). We used the recommended threshold of ≥95% ANI to delimit the boundaries between species (Jain et al., 2018). We used the mob-recon v.3.1.0 tool from MOB-suite (Robertson and Nash, 2018) to identify and characterize the plasmids.

ABRicate v.1.0.0^1^ was used as a screening tool to align the sequenced genomes and plasmid sequences to the CARD database (Alcock et al., 2023) to identify the presence and location of the *bla*TEM gene, using coverage values >60% and identity >80%. The Resistance Gene Identifier (RGI; Alcock et al., 2023) was then used to confirm the presence of the *bla*TEM gene and *β*-lactamase families in the sequenced genomes. The parameters adopted included the DIAMOND aligner and the *include_loose* flag, and genes with identity values >80% were considered positive. The plasmid sequences were annotated using Bakta (Schwengers et al., 2021), and the GenBank files generated were used as input for Clinker (Gilchrist and Chooi, 2021), to compare sequences and locate the *bla*(TEM) gene.

Statistical analysis of the results. Statistical analysis of the results. The data was tabulated, *log-transformed* and subjected to normality analysis. One way ANOVA with a 5% of significance level was used to compare the tests and possible differences in the values obtained in Traditional/No supplement, Whey or chicken juice. The T-test was used to compare groups with and without Lu54 treatment. All analyses were carried out using the *GraphPad Prism* 8.0 program (GraphPad Software Inc., San Diego, CA, United States).

## 3 Results

Conjugation frequency—different methodologies. The counts of donor, recipient and transconjugant bacteria in all these repetitions showed no statistical difference between them (*p* < 0.05—one way ANOVA), thus guaranteeing the quantitative maintenance of each test.

**GRAPH 1:**
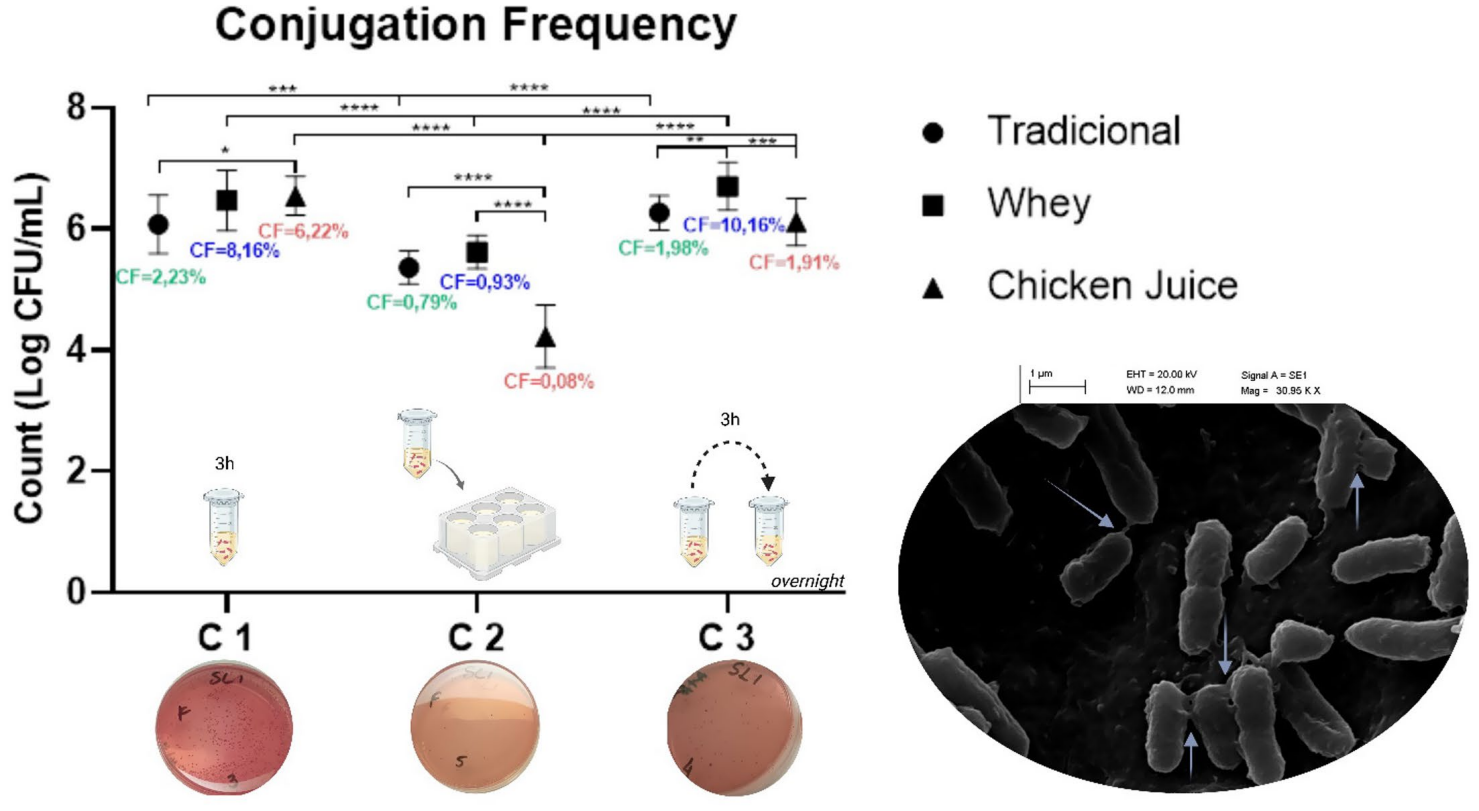
Conjugation frequencies in the different methodologies. C1: Conjugation 1, C2: Conjugation 2 and C3: Conjugation 3. *p < 0.05, **p < 0.01, ***p < 0.001, ****p < 0.0001 (one way ANOVA). The graph mentions the methodology used in each type of conjugation, as well as the phenotypic demonstration of the presence of *E. coli* transconjugants on MacConckey plates. The scanning electron microscopy (SEM) image of bacterial conjugation between *Salmonella* Heidelberg and *Escherichia coli* (on the right) demonstrates multiple pili (arrows) for horizontal gene transfer (image obtained at 30,000 × and 40,000 × magnifications).

When comparing the results obtained in the three protocols (GRAPH 1), we found that conjugation 1 was the most effective, since the conjugation frequency (RCF) was 2.23%, to the detriment of 0.79 and 1.98% identified in methods 2 and 3, respectively. At the same time, we found that technique 1 was also more promising in the presence of WH and *CJ*, whose percentages of RCF were equivalent to 8.16 and 6.22%, respectively. The exception was method 3, which was the most effective in the presence of WH (10.16%).

Statistical analysis revealed significant differences in all three conjugation groups. In Conjugation 1, there was a difference between the traditional (T) and CJ groups (*p* = 0.0436). In Conjugation 2, significant differences were found between T and CJ (*p* < 0.0001) and between WH and CJ (*p* < 0.0001). For Conjugation 3, differences were observed between T and SL (*p* = 0.0057) and between SL and CJ (*p* = 0.00070). Inter-conjugation comparisons were also conducted. Significant differences were observed between Conjugation 1 and 2 for the T, SL, and CJ groups (*p* = 0.0001, *p* < 0.0001, and *p* < 0.0001, respectively). Likewise, Conjugations 2 and 3 showed significant differences across all three groups (T, SL, and CJ), with all *p*-values less than 0.0001. No significant statistical difference was found between Conjugations 1 and 3.

We found that WH increased gene recombination rates, especially in liquid media, in conjugations 1 and 3, with values equivalent to four and five orders of magnitude, respectively. *CJ*, on the other hand, increased the rates by three orders of magnitude for conjugation in liquid with 3 h of incubation.

**GRAPH 2:**
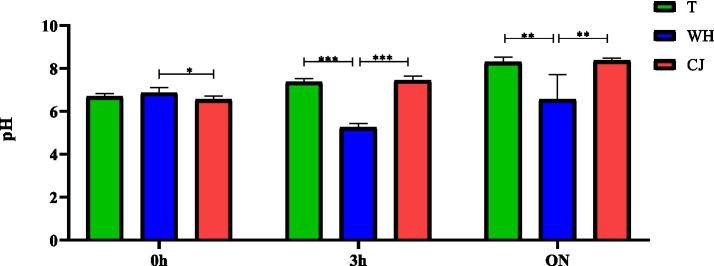
pH variation of the conjugation media in liquid at the different incubation times. T = traditional, with Luria Bertani (LB). WH = LB + 5% whey. CJ = LB + 5% chicken juice. 0 h: Immediately after donor and recipient were joined (start of conjugation). 3 h: End of conjugation 1. ON = *overnight* (End of conjugation 3). *p < 0.05, **p < 0.01, ***p < 0.001 (one way ANOVA). Different uppercase letters in the same row indicate statistical difference by ANOVA test (*p* < 0.05); different lowercase letters in the same column indicate statistical difference by ANOVA test (*p* < 0.05); T, Traditional/No supplement; WH, Whey; CJ, chicken juice. Conjugation 1: Liquid medium, incubated at 37 °C, for 3 h, 20 rpm; Conjugation 2: Solid medium, incubated at 37 °C, *overnight*, without stirring; Conjugation 3: Liquid medium, incubated at 37 °C, *overnight*, without stirring.

It is worth considering that the incubation time in the liquid conjugation media influenced the pH indices, especially in the presence of WH (GRAPH 2). There was a clear reduction in the pH of the medium with WH, varying from an average pH of 7.4 in the Traditional (media without *CJ* and WH) and *CJ* tests to 5.2 in WH (*p* < 0.0001, one way ANOVA) after 3 h and from 8.32 in the Traditional and CJ tests to 6.5 when supplemented with WH (*p* = 0.0005, one way ANOVA) in *overnight* incubation.

Table 1 shows the average counts (log CFU/mL) obtained in the conjugation protocols in all the tests. We found that in conjugation 1 we obtained an average increase of 0.4 and 0.5 log CFU/mL (*p* = 0.0130) when in the presence of WH and CJ. In methods 2 and 3, the increase was only identified in the presence of WH and was equivalent to averages of 0.3 and 0.4 log UFC/mL (*p* = 0.0444 and *p* > 0.05), respectively. In general, conjugation 2 was statistically the least effective in the production of transconjugants both in the traditional test with LB and with the supplementation of WH and CJ (*p* < 0.0001).

**Table 1:** Mean count (Log CFU/mL) of donor, recipient, and transconjugant bacteria and conjugation frequency obtained in the three conjugation protocols, with and without the addition of 5% whey and chicken juice.

Strains	Conjugation 1 (log CFU/mL)	Conjugation 2 (log CFU /mL)	Conjugation 3 (log CFU /mL)
Donor (*S*H)	7,60 ± 0,21^Aa^	7,42 ± 0,47 ^Aa^	7,73 ± 0,32 ^Aa^
Recipient (J53)	7,63 ± 0,34^Ba^	7,50 ± 0,12 ^Ba^	7,76 ± 0,33 ^Ba^
Transconjugants (T)	6,08 ± 0,48 ^Cb^	5,37 ± 0,28 ^Db^	6,27 ± 0,28 ^Cb^
Transconjugants (WH)	6,47 ± 0,50^Ebc^	5,62 ± 0,27 ^Fb^	6,71 ± 0,40 ^Ec^
Transconjugants (CJ)	6,54 ± 0,32^Gc^	4,23 ± 0,52 ^Hc^	6,11 ± 0,39 ^Gb^

Different uppercase letters in the same row indicate statistical difference by ANOVA test (*p* < 0.05); different lowercase letters in the same column indicate statistical difference by ANOVA test (*p* < 0.05); T, Traditional/No supplement; WH, Whey; CJ: Chicken juice. Conjugation 1: Liquid medium, incubated at 37 °C, for 3 h, 20 rpm; Conjugation 2: Solid medium, incubated at 37 °C, overnight, without stirring; Conjugation 3: Liquid medium, incubated at 37 °C, overnight, without stirring.

Treatment with the Lu54 copper complex. The tests to determine the best concentration of the Lu54 copper complex allowed us to identify that the minimum inhibitory concentration (MIC) was equivalent to 250 ug/mL, while the concentration of 125 ug/mL was the most effective for conjugation, since there was no significant difference in reducing the count of *E. coli* (*p* = 0.42) or *Salmonella* Heidelberg (*p* = 0.46), and thus did not interfere with the results of the conjugations. At this concentration, we had a count of 6.71 ± 0.30 log UFC/mL in the control and 6.84 ± 0.37 log UFC/mL in the test with Lu 54 in *E. coli*, while in the test with *S.* Heidelberg we had 6.81 ± 0.58 log UFC/mL and 7.00 ± 0.52 log UFC/mL in the control and test, respectively.

**GRAPH 3:**
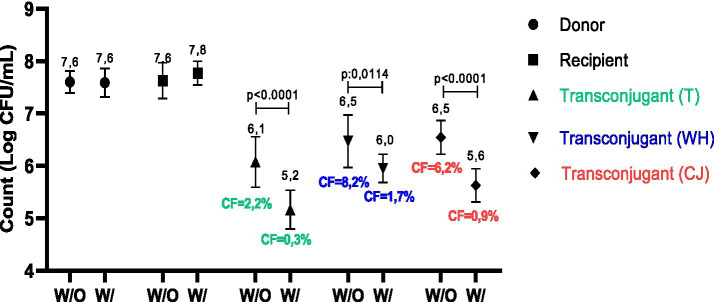
Comparison of counts and conjugation frequency obtained in conjugates with and without treatment with 125 μg/mL Lu54 [Cu(Clmp)(mTFpy)](PF_6_)_2_. W/O, Without Lu54 treatment; W/, With Lu54 treatment; T, traditional; WH: whey; CJ, chicken juice. CF, Conjugation frequency in percent. *p* < 0.0001 and *p* = 0.0114, statistical difference by T-test when comparing with and without Lu54 treatment.

Conjugation method 1 was selected for the tests with Lu54 due to its effectiveness in producing transconjugants. The use of this copper complex showed efficiency in controlling the conjugation process since there was a reduction in RCF from 2.2 to 0.3% in the traditional test, from 8.2 to 1.7% in conjugation with WH and from 6.2 to 0.9% in the presence of CJ, equivalent to a 7.3, 4.8 and 6.9-fold reduction, respectively. The values found also showed a statistical difference in transconjugant counts from 6.1 to 5.2 log UFC/mL in traditional conjugation, 6.5 to 6.0 log UFC/mL in the presence of WH and 6.5 to 5.6 log UFC/mL with CJ supplementation (GRAPH 3).

The NCBI accession numbers for the deposited genome sequence data are provided in the Data Availability Statement. Whole genome sequencing (WGS). The six sequenced genomes had between 2 and 5 contigs (mean 3.83), with a total size between 4706500pb and 4778038pb (mean 4743274.67pb), completeness between 96.12 and 97.57% (mean 96.85%) and contamination between 0.38 and 0.69% (mean 0.53%), within the quality metrics (Supplementary Table 1). The species was confirmed, with the genomes showing >99% average nucleotide identity when compared to the reference genome.

The *bla*TEM-181 gene was identified in three genomes by Abricate, which were not treated with Lu54. RGI analysis identified *bla*TEM variants (*bla*TEM-1, *bla*TEM-91, *bla*TEM-98, *bla*TEM-144, *bla*TEM-186, *bla*TEM-219, *bla*TEM-232). The pipeline confirmed the results, and separated the transconjugants into two distinct clusters based on the presence of Lu54, showing that the transfer of *bla*TEM occurred only in the untreated samples, while the beta lactamase *ampC* was identified in all the transconjugants (Supplementary Table 2).

**Figure 1:**
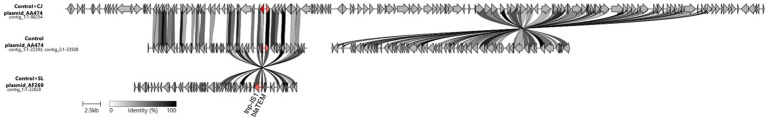
Comparison of plasmid contigs in which the *bla*TEM gene was identified. Control = control without the addition of Lu54; CJ = chicken juice; SL = whey; Tnp-IS1 = transposable element flanking the *bla*(TEM) gene. The colors of the bars linking the genes are based on the percentage of identity between the genes.

Fourteen plasmids were identified in the sequenced genomes, with each genome having between one and three plasmids (Supplementary Table 3). The *bla*TEM gene was identified in three plasmids, which were in the genomes of control *E. coli* (without CJ, WH and Lu54), control + CJ and control + WH. These plasmids were annotated with Bakta (Schwengers et al., 2021), and compared using clinker (Gilchrist and Chooi, 2021), to visualize *bla*TEM (Figure 1).

## 4 Discussion

The increase in antimicrobial resistance (AMR) has become a global public health problem, which makes it urgent to search for more in-depth knowledge about the mechanisms that affect and stimulate this phenomenon, as well as the search for new control alternatives (Frieri et al., 2017). In this context, conjugation is the most common form of recombination and dissemination of AMR in bacteria and is caused by the transfer of a gene from the donor bacterium to the recipient through direct contact (Neil et al., 2021). Transfer by conjugation can occur at different times, in different media and on different surfaces, such as the intestinal microbiota (Neil et al., 2021), soil (Tecon et al., 2018), aquatic conditions (Arias-Andres et al., 2018) and microbial biofilms (Águila-Arcos et al., 2017). There is no defined methodology for determining resistance gene transfer by conjugation, which prompted this investigation into the differences in *bla*TEM transfer in both liquid (at different times) and solid media.

Conjugation in liquid media probably favors greater exposure and immediate contact between the bacteria, which enhances the process, as we identified in method 1, which was the most efficient. This method also differed in that it used agitation, which, in addition to aeration, can improve access to nutrients and increase the frequency of collisions cell, as shown by Headd and Bradford (2018), who also observed that, in general, there was a higher frequency of conjugation in agitated cultures than in non-agitated cultures, although vigorous agitation can cause the conjugative pili to break. However, maintaining these bacteria overnight and removing moderate agitation (conjugation 3) can reduce the conjugation frequency (CF) due to extrinsic and intrinsic oscillations resulting from the maintenance medium, metabolism and bacterial physiology, such as stress caused by increased osmolarity, reduced nutritional availability and changes in the pH of the medium (Saliu et al., 2019; Kohyama and Suzuki, 2019; van Wonterghem et al., 2022).

We found that the average conjugation rates in *solid/overnight* media were lower than 1% and therefore lower than the other methods. By fixing the bacteria in a space/membrane, conjugation is restricted to neighboring cells and cell motility is reduced when compared to liquid media, which can hinder the frequency of gene exchanges. In addition, in relation to the incubation period, our results corroborate previous studies which found significant reductions in CF in experiments with a contact time of between 16 and 24 h compared to those with a time of less than 4 h (Alderliesten et al., 2020; Sakuda et al., 2018).

In addition to the differences between the times and media used, it is important to establish the interference that the presence of supplements can cause in the conjugation process. This study is one of the first to demonstrate the influence of the presence of whey and chicken juice - by- products routinely found in animal product processing industries - on bacterial conjugation.

In Brazil, this issue is even more relevant, considering that agriculture is one of the most important economic activities in the country, which ranks third in the world in meat production and second largest exporter, in addition to significant milk production (EMBRAPA, 2021). This role highlights the need to produce innocuous foods and to better understand the influence of by-products commonly present in the meat and cheese processing industries on the transmission and consequent spread of resistance genes.

In this context, WH and CJ were shown to enhance the transfer of the *bla*TEM gene by conjugation in liquid media. Both of the by-products used in this study are rich in nutrients, such as carbohydrates, lipids and proteins (Melo et al., 2017; Prazeres et al., 2012) which are widely used by bacteria for their metabolism (Kim and Gadd, 2008; Alderliesten et al., 2020), which certainly provides greater multiplication, survival and conditions to allow these microrganisms to express their potential. However, other circumstances can also influence the action of bacteria, such as incubation time, cell density, species used, the ratio between donor and recipient, temperature and changes in pH (Alderliesten et al., 2020).

Pallares-Vega et al. (2021) found that temperatures of 25 °C or less and low nutrient media can reduce the number of conjugative events. A similar situation occurs when there is an increase in the density of donors in relation to recipients (Chatterjee et al., 2013). The fact that the donor and recipient bacteria belong to the same species, when in a 1:1 ratio, both in liquid and filter media, can lead to an increase in the frequency of plasmid transfer. The same study also observed that, although the frequency of transfer was similar at both three and 16 h, the trend was lower in the longer period (Sakuda et al., 2018).

With regard to the influence of pH on the conjugation process, our study made it clear that reducing it to values between 5.2 and 6.5 in the presence of WH intensified the transfer of the *bla*TEM gene, while alkalinization caused lower production of transconjugants. In broth containing pH 5.0, at a temperature of 37 °C, the microorganisms obtain partial resistance to the acid in 5 mins and complete resistance in 10 min, which does not occur at a pH higher than 6.5. This habituation requires the synthesis of proteins and, when compared to organisms grown at pH 7.0, those grown at pH 5.0 have greater quantities of proteins, especially cytoplasmic proteins, which may be involved in protecting the cell against acid damage or in DNA repair (Raja et al., 1991). On the other hand, pH close to 8.0 can characterize a condition of alkaline stress, which in a study conducted by Cortés et al. (2016) represented a 36% decrease in the specific growth rates of *E. coli* when compared to those at pH 7.2. In addition, Van Wonterghem et al. (2022) pointed out that lowering the pH (from 7.6–8.0 to ~7.4) can boost conjugation. They also point out that pH dependence has been observed in the transfer of some plasmids, with pHs of around 6.0–7.5 being considered optimal for conjugation.

Also in this regard, the fact that the transfer of the *bla*TEM gene in liquid media containing whey is at least three times greater than in its absence is even more worrying, since dairy farms use antimicrobial treatments constantly, which can lead to microorganisms present in milk showing high levels of resistance. WH is abundantly present in many dairy products and it is also possible to infer that raw or pasteurized milk may exert the same tendency to increase conjugation rates. In agreement with this concern, a study conducted in China analyzing 350 raw milk samples from dairy farms found *E. coli* in 23.1% of the samples, with 33.3% of the isolates classified as multidrug resistant (MDR). Antimicrobial susceptibility testing revealed resistance to 92.9% of the 14 antibiotics tested, except meropenem, and notably, the *bla*TEM gene was present in 92.6% of the MDR isolates (Gao et al., 2025).

In addition, the use of raw milk for cheese making is a common practice, and the lack of heat treatment allows a greater number of bacteria, including those from dairy animals and the milking environment, to remain viable, which consequently amplifies the presence of resistance genes and increases the risk of horizontal gene transfer (Tóth et al., 2020; Marques et al., 2020). Supporting this, a study on small farms in Brazil detected pathogenic, commensal, and ceftriaxone-resistant (ESBL) *E. coli* at various points in milk collection and cheese production, including pathogenic strains of EPEC, STEC, and ExPEC. Where ExPEC or potentially ExPEC carried the *bla*TEM gene. Many isolates were resistant to antimicrobials such as nalidixic acid, ampicillin, kanamycin, streptomycin, sulfisoxazole and tetracycline. Genetic analysis revealed the presence of identical virulence genes at different stages of production within the same farm (Ribeiro et al., 2024).

At the same time, CJ also proved to enhance the process of gene exchange by conjugation, just as it had already intensified other bacterial perpetuation processes, such as the formation of biofilms. Melo et al. (2017) found that supplementation with CJ allowed viable cells to be maintained and strong biofilms to form, supporting the idea that this by-product promotes the survival and permanence of microorganisms in the production chain and can play an important role in food safety.

In addition, it is necessary to consider that the influence of these by-products on gene transfer can be considered a unique health problem, since, if not properly treated in the industry, the disposal of this waste in the environment further amplifies the spread of AMR in an environmental context, since animals, soil and aquatic organisms also contribute to the origin, maintenance and propagation of this phenomenon (Berendonk et al., 2015). In view of the above, it can be seen that horizontal gene transfer and antimicrobial resistance are global public health problems that can be exacerbated in conditions where animal products are processed. The emergence of antimicrobial resistance has led to difficulties in providing effective therapies and a lack of successful industrial hygiene prevention measures, which requires the development of new alternatives that can also prevent AMR (Frieri et al., 2017). In this way, we present an option for controlling the *bla*TEM transfer process using a copper complex.

In general, the antimicrobial potential of copper can occur through the generation of reactive oxygen species (ROS), which compromise proteins and DNA, or through oxidative damage to phospholipids in the bacterial membrane. In addition, these metals can interfere with the functioning of enzymes by competing with or displacing other ions needed in the active site or by binding covalently to functional groups in the active site, thus preventing their function and maintaining bacterial viability (Palm et al., 2022; Zhang et al., 2019).

In addition, the use of copper complexes can reduce or promote conjugation events. We found that the use of Lu54 was able to reduce the transfer of the *bla*TEM gene by conjugation at least 4.8 times, both in the presence and absence of supplements. These results were confirmed by whole genome sequencing analysis, where plasmids containing the *bla*TEM gene were identified only in control samples with or without supplements, but not in the presence of Lu54. Our results are corroborated by Buberg et al. (2020), who investigated the influence of copper on the conjugative transfer of a plasmid carrying the *bla*CMY-2 gene isolated from extended-spectrum betalactamase (ESBL)-producing *E. coli* from retail chicken meat, and found that there was at least a 40% reduction in transfer when compared to the control. They justified the reduction by the presence of copper, which is capable of causing disturbances in the function of the donor’s conjugative machinery by reducing the gene expression of *traB* and *nikB*, two important genes responsible for the ability to mobilize and transfer plasmids from one strain to another. However, these results contrast with a study that used *E. coli* K-12 as the donor, carrying the RP4 plasmid, which is resistant to kanamycin, ampicillin and tetracycline, and *Pseudomonas putida* KT2440 as the recipient. The use of Cu^2+^ led to greater production of ROS, expression of proteins involved in oxidative stress and increased permeability of the donor and recipient membranes, which promoted greater gene transfer by conjugation (Zhang et al., 2019). These differences are probably due to different metal concentrations and compositions, differences in experimental conditions, bacterial species and associated plasmids, as well as genes and proteins involved in conjugation.

WGS-supported analysis proved effective in differentiating the mobilome and families of *β*-lactamases, as well as highlighting the selective impact of Lu54 on mobile elements. The reduction in CF and the disappearance of *bla*TEM after treatment suggest that copper interferes with both the transfer machinery and the stability of plasmids, confirming its potential as a sanitary barrier in industrial environments. Our results revealed that by-products of animal origin favor the spread of *bla*TEM, while the Lu54 complex acts to reduce this spread and reorganize the plasmid resistome.

The difference in the *bla*TEM gene variants identified by Abricate and RGI demonstrates the greater specificity of the latter, which discriminated several variants based on specific mutations associated with distinct resistance profiles. In contrast, Abricate grouped these highly similar sequences under a single reference (*bla*TEM-181)The discrepancy between variants observed in RGI may be related to two main factors: (i) the emergence of variants during the experimental treatments with CJ and WH, or (ii) technical limitations inherent in the quality of the sequenced genomes, since it has already been shown that the quality of the data and the assembly method significantly interfere with the accurate identification of point mutations (Zankari et al., 2017). Despite these differences, both methods were concordant in identifying the *bla*TEM gene, confirming the presence of this family of β-lactamases in isolates not treated with Lu54.

From the results, it was concluded that the presence of whey and chicken juice, which mimic industrial conditions, is capable of stimulating the transfer of the *bla*TEM gene by conjugation, and the methodology using liquid medium, a few hours of incubation and agitation is the most effective for this event to occur. Furthermore, the use of the [Cu(Clmp)(mTFpy)](PF_6_)_2_ complex has proved to be a promising alternative for controlling gene exchange and mitigating antimicrobial resistance, especially in industrial environments where animal products are processed.

## Data Availability

Genome sequence data of the six *E. coli* isolates sequenced in this study are available in the National Center for Biotechnology Information Assembly/Genome database under BioProject accession number PRJNA1344634, biosamples SAMN52649862, SAMN52649863, SAMN52649864, SAMN52649865, SAMN52649866, SAMN52649867.

## Footnotes

^1^ https://github.com/tseemann/abricate
